# pH-Responsive Hydrogel as a Potential Oral Delivery System of Baicalin for Prolonging Gastroprotective Activity

**DOI:** 10.3390/pharmaceutics15010257

**Published:** 2023-01-11

**Authors:** Lixing Xu, Enhe Bai, Yangbo Zhu, Jiayi Qin, Xiao Du, Haiqin Huang

**Affiliations:** 1 Department of Pharmaceutics, School of Pharmacy, Nantong University, Nantong 226001, China; 2 Xiangya International Academy of Translational Medicine, Central South University, Changsha 410013, China; 3 Department of Surgery, Division of Hepatobiliary and Pancreatic Surgery, The First Affiliated Hospital, Zhejiang University School of Medicine, 79 QingChun Road, Hangzhou 310003, China; 4 Department of Pharmacy, Nanjing Drum Tower Hospital, The Affiliated Hospital of Nanjing University Medical School, Nanjing Medical Center for Clinical Pharmacy, Nanjing 210008, China

**Keywords:** gastric ulcer, pH-responsive in situ gelation, baicalin, sustained release, oxidative stress

## Abstract

Gastric ulcer is one of the most common gastrointestinal diseases, and natural products have obvious advantages in the treatment of gastrointestinal diseases. Baicalin (Bai) extracted from scutellaria baicalensis exhibits anti-inflammatory, antioxidant, and anti-apoptotic activities. Herein, a pH-responsive sodium alginate/polyaspartate/CaCO_3_ (SA/PASP@CaCO_3_) in situ hydrogel was established for the oral delivery of Bai. In this study, we detected the gelling properties, mechanical strength, in vitro erosion, and in vitro release behavior of the hydrogels. Meanwhile, the efficiency of Bai/SA/PASP@CaCO_3_ hydrogel on ethanol-induced acute gastric ulcers, acetic acid-induced chronic gastric ulcers, and H_2_O_2_-stimulated human gastric epithelial GES-1 cells was explored. The pathological examination revealed that Bai-loaded hydrogel alleviated acute and chronic gastric ulcers. In vivo and in vitro results further confirmed that Bai/SA/PASP@CaCO_3_ in situ hydrogels significantly relieved oxidative stress injury. Moreover, through Western blot assay, Bai/SA/PASP@CaCO_3_ hydrogel was also found to dramatically increase the proteins levels of NRF2, HO-1, and Bcl2, and reduce levels of p-JNK, cleaved-caspase-3 and Bax; through flow cytometry, it was observed to significantly inhibit the H_2_O_2_-induced apoptosis of GES-1 cells. Importantly, the Bai/SA/PASP@CaCO_3_ in situ hydrogel system showed better anti-gastric ulcer efficiency than free drug, and could serve as a potential drug delivery system for the clinical treatment of gastric ulcers.

## 1. Introduction

Gastric ulcer (GU) is a common benign gastrointestinal disease in humans. It has become a major threat to public health, and a burden on health care globally [1,2]. Currently, the existing clinical regimen of histamine 2 receptor antagonists, proton pump inhibitors, and H. pylori clearance is mostly adopted for the treatment of GU [3,4]. Although current therapy has achieved some efficacy, it still has the following drawbacks: (1) the systemic use of antibiotics is likely to cause organism resistance; (2) the target of the combination treatment strategy commonly used is relatively poor, and high doses of frequent drug administration are prone to cause toxic side effects; (3) the relapse rate is high after discontinuation. Therefore, it is still necessary to find a targeted treatment strategy. The occurrence and progression of GU have been reported to be associated with oxidative stress [3,4,5]. Research has confirmed that stimulation of the gastric mucosa by Helicobacter oxytoca, gastric acid, and alcohol consumption can cause oxidative stress, which leads to DNA damage and necrosis, or apoptosis of gastric mucosal epithelial cells [6]. Therefore, effective inhibition of oxidative stress at the site of gastric ulcers could inhibit the progression of gastric ulcer disease.

Bai, as an active ingredient of herbaceous plants, possesses good pharmacological effects such as anti-ulcer, anti-oxidative stress, anti-inflammatory, and promoting wound healing [7,8,9,10,11,12,13]. As reported, Bai could protect against LPS-induced blood–brain damage via activating the Nrf2-mediated antioxidant stress pathway [14]. Meanwhile, Bai could reduce ethanol-induced liver oxidative damage by regulating the NRF2/HO-1/NLRP3 pathway [15,16]. Thus, it is clear that Bai can be selected as an effective therapeutic agent against oxidative stress. Despite its excellent medicinal effect, the extremely low solubility of Bai leads to a lower bioavailability in vivo, thus limiting its application in treatment [17,18]. Therefore, a suitable delivery system is urgently required to elevate the bioavailability of Bai, and improve its efficacy.

Hydrogel, with its three-dimensional cross-linked structure, has an environment similar to that of the natural extracellular matrix, allowing for exchange with body fluids, and providing nutrients for cells and tissues [19,20]. Therefore, hydrogels, with their good biosafety and histocompatibility, have received increasing attention [21]. Intragastric pH-responsive hydrogels could linger and adhere to the stomach for prolonged retention and enhanced efficacy [22]. Sodium alginate (SA), a polypolysaccharide material with good biocompatibility, mucosal adhesion, and biodegradability, is an excellent candidate for gastric retention delivery systems. The aqueous solution of SA can undergo gelling reactions in the presence of divalent ions (Ca^2+^, Cu^2+^, Mn^2+^, etc.) [23]. Commonly, Ca^2+^ is the most used cross-linking agent due to its excellent biocompatibility; it can also chelate with multiple oxygen atoms in the sodium alginate structure to form an “egg box” structure [23]. However, the addition rate of Ca^2+^ affects the morphology and the ionic cross-linking strength of the resulting hydrogel. Therefore, CaCO_3_, which can be soluble at lower pH conditions, is commonly investigated as an effective Ca^2+^ source for SA hydrogels. As reported, ACC/SA in situ gel sheet was constructed by Xu et al. for the oral delivery of diclofenac sodium, achieving sustained release and enhanced bioavailability [24].

**Scheme 1:**
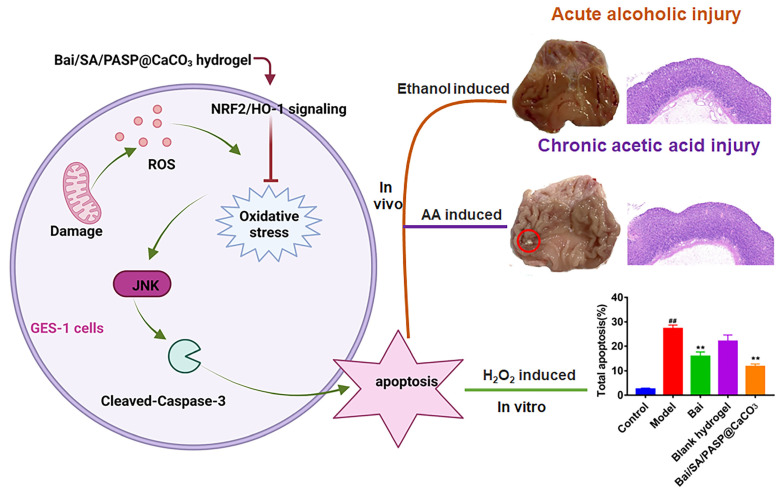
Illustration of Bai-loaded SA/PASP@CaCO_3_ hydrogel applied for the treatment of three GU models by inhibiting oxidative stress through the NRF2/HO-1 signaling pathway. Significant difference from the control group: ^##^ *p* < 0.01; Significant difference from the model group: ** *p* < 0.01.

Thus, an in situ sodium alginate/polyaspartic acid/CaCO_3_ hydrogel (SA/PASP@CaCO_3_) with gastric acid pH responsiveness for sustained release of Bai was designed for alleviating oxidative stress damage in gastric ulcer disease (Scheme 1). PASP, which is commonly used as a green scale inhibitor, was selected to form stable CaCO_3_ nanoparticles (NPs), leading to uniform dispersion in the SA solution and improved homogeneity of the formed hydrogel. With the continuous release of Bai in the gastric lining, it was effectively absorbed, thus preventing GU by inhibiting oxidative stress through the Nrf2/HO-1 signaling pathway, both in vitro and in vivo. These findings suggest that the in situ hydrogels delivery system can serve as a potential vector for the oral delivery of Bai, thereby achieving effective treatment of GU in clinical settings.

## 2. Materials and Methods

### 2.1. Materials

Sodium alginate (SA, 200~400 mPa.s) was supplied by Luyuan Biotechnology Co., Ltd. (Shandong, China). Polyaspartic acid (PASP) and sodium bicarbonate anhydrous were provided by Nanjing Chemical Reagent Co., Ltd. (Nanjing, China). Anhydrous calcium chloride was obtained by Energy Chemical Technology Co., Ltd. (Shanghai, China). Baicalin (Bai) was obtained from Sigma-Aldrich Co., Ltd. (St. Louis, MO, USA). Annexin V FITC/PI Apoptosis Kit, fetal bovine serum (FBS), Dulbecco’s modified eagle medium, streptomycin, and penicillin were obtained from Beyotime Biotechnology Co., Ltd. (Shanghai, China). H_2_O_2_ was purchased from Aladdin (Shanghai, China). Nuclear factor erythroid 2 (NRF-2, 12721) antibody, Heme oxygenase-1 (HO-1, 43966) antibody, Jun N-terminal kinase (JNK, 9252) antibody, phospho-JNK (p-JNK, 4668) antibody, cleaved caspase-3 (9664) antibody, Bcl-2 associated X (BAX, 14796) antibody, and B cell lymphoma-2 (Bcl-2, 3498) antibody were provided by CST (MA, USA). The assay kits of Malondialdehyde (MDA, A003), Superoxide dismutase (SOD, A001), Catalase (CAT, A007), and Glutathione (GSH, A006) were purchased from Jiancheng (Nanjing, China).

### 2.2. Preparation and Characterization of PASP@CaCO_3_ NPs

PASP@CaCO_3_ NPs were synthesized according to the one-pot strategy [24]. The preparation process is described below. First, 100 mg of anhydrous calcium chloride was dissolved in 10 mL of deionized water, followed by the addition of 1 mL of 40% (*w/w*) PASP solution, and stirred continuously at 200 rpm for 1.5 h at 37 °C. Subsequently, 15 mL of 0.7% (*w/v*) sodium carbonate solution was added dropwise to the above mixture solution and stirred continuously at 200 rpm for 20 min. Finally, the above solution was centrifuged at 9000 rpm for 5 min, and the supernatant was discarded to collect PASP@CaCO_3_ NPs. The PASP@CaCO_3_ NPs were washed with deionized water twice, and then resuspended by adding an appropriate amount of deionized water before being stored in a refrigerator at 4 °C.

The particle size distribution and zeta potential of PASP@CaCO_3_ NPs were detected using dynamic light scattering through a laser particle size analyzer (Malvern Zetasizer Nano ZS90, Malvern, UK). The morphology was observed through transmission electron microscopy (TEM, JEOL, Japan). Moreover, the structure of the PASP@CaCO_3_ NPs was characterized via Fourier transform infrared spectrometry (FT-IR, JASCO, Japan).

### 2.3. Preparation of SA/PASP@CaCO_3_ Hydrogels

First, 2.0 g of SA was weighed into 100 mL of deionized water to prepare a 2% (*w/v*) SA solution. Then, 5 mL of the above-prepared PASP@CaCO_3_ NP solution was added to 5 mL of SA solution dropwise, and mixed well to form the pre-gel solution. In order to investigate the gelation properties of the pre-gel solution, pH 1.2 HCl solution was added dropwise to the pre-gel solution, and the gel formation process was photographed and recorded.

Since Bai is insoluble in water, we adopted ethanol as a co-solvent to prepare Bai solution. Briefly, 20 mg of Bai was weighed into 1 mL of 50% (*v/v*) ethanol solution to prepare Bai solution. The prepared Bai solution was uniformly dispersed in 2% SA solution to form Bai/SA solution (the concentration of Bai was 4 mg/mL), and then the same volume of PASP@CaCO_3_ nanoparticles was added to SA solution, forming a Bai/SA/PASP@CaCO_3_ pre-gel solution. The Bai-loaded SA/PASP@CaCO_3_ hydrogel was formed by adding pH 1.2 HCl solutions to the pre-gel solution. In order to detect the drug loading and encapsulation efficiency of Bai, Bai/SA/PASP@CaCO_3_ hydrogels were immersed in 50 mL of 50% ethanol solution, and stirred continuously at 400 rpm at 37 °C until the hydrogel completely dissolved and disappeared. The concentration of Bai in the solution was determined using high-performance liquid chromatography (HPLC, LC-20AT, Shimadzu, Japan). The high-performance liquid chromatography (HPLC) conditions were as follows: detection wavelength, 278 nm; column, C18; mobile phase, methanol–water-phosphoric acid (49:51:0.2); injection volume, 20 µL; flow rate, 1 mL/min; column temperature, 28 °C. In addition, a standard curve for Bai was established to determine the concentration of Bai. Drug loading and encapsulation efficiency were calculated according to the following formulas:

$$\mathrm{Drug}\;\mathrm{loading}\left(\%\right)=\frac{m_{\mathrm{Bai}\;\mathrm{in}\;\mathrm{hydrogel}}}{m_{\mathrm{hydrogel}}}\times 100\%$$

$$\mathrm{Encapsulation}\;\mathrm{efficiency}\left(\%\right)=\frac{m_{\mathrm{Bai}\;\mathrm{in}\;\mathrm{hydrogel}}}{m_{\mathrm{Bai}\;\mathrm{added}}}\times 100\%$$

### 2.4. Characterization of SA/PASP@CaCO_3_ In Situ Hydrogel System

#### 2.4.1. Linear Viscoelastic Region Determination

SA/PASP@CaCO_3_ hydrogels were prepared according to Section 2.3. The variation curves of the storage modulus (G′) and loss modulus (G″) of SA/PASP@CaCO_3_ hydrogels were measured at a fixed frequency (parallel plate diameter of 40 mm) using the strain scan mode of DHR-2 rotational rheometer (TA Instruments-waters LLC, New Castle, DE, USA).

#### 2.4.2. Effect of pH Variation on Gelling Capacity

To assess the effect of pH variation on the gelling capacity, solutions with pH 1.2, 3.0, and 4.5 were added dropwise to the SA/PASP@CaCO_3_ pre-gel solutions prepared above. Next, variation curves of the storage modulus (G′) and loss modulus (G″) of SA/PASP@CaCO_3_ in situ hydrogel fragments obtained in different pH solutions were determined using the frequency scan mode of a DHR-2 rotational rheometer (TA Instruments-waters LLC, New Castle, DE, USA) (parallel plate diameter of 40 mm and oscillation strain of 2%).

#### 2.4.3. Weight Loss Assay of SA/PASP@CaCO_3_ Hydrogel

The weight loss assay was carried out in pH 1.2 HCl medium to detect the erosion and degradation of Bai/SA/PASP@CaCO_3_ in situ hydrogels. In brief, the SA/PASP@CaCO_3_ hydrogel was weighed and noted as the initial mass (m_0_). The hydrogel was then immersed in pH 1.2 HCl solution and continuously shaken at 100 rpm and 37 °C. After that, the hydrogels were taken out at 2, 4, 6, 12, 16, 24, 36, and 48 h, and weighed as m_t_. The weight loss was calculated with the following equation:

$$\mathrm{Weight}\;\mathrm{loss}\left(\%\right)=\frac{m_{0}-m_{t}}{m_{0}}\times 100\%$$

#### 2.4.4. In Vitro Drug Release of Bai from Bai/SA/PASP@CaCO_3_ Hydrogels

The in vitro drug release profile of the Bai/SA/PASP@CaCO_3_ hydrogels was assessed via the dialysis bag diffusion method. A volume of 5 mL of pre-gel solution containing 2 mg/mL Bai was placed in a dialysis bag (MWCO = 8000 Da), which was then immersed in 30 mL of HCl solution at pH 1.2 (containing 50% ethanol). Bai was released by continuously stirring at 100 rpm at 37 °C. Samples of 2 mL were taken at 1, 2, 3, 4, 5, 6, 7, 8, 12, 16, 24, 36, and 48 h, and supplemented with an equal volume of pH 1.2 HCl solution (containing 50% ethanol). The concentration of Bai was detected with the HPLC method described above. The released percent of Bai was calculated according to the following formula:

$$\mathrm{Drug}\;\mathrm{release}\;\mathrm{percent}\left(\%\right)=\frac{C_{n}{\times V}_{n}{+C}_{n-1}{\times V}_{n-1}{+C}_{n-2}{\times V}_{n-2}{+\ldots +C}_{1}{\times V}_{1}}{M_{\mathrm{Bai}}}\times 100\%$$

where C_n_ and C_n−1_ were the concentrations of Bai released at times n and n − 1, respectively. V_n_ and V_n−1_ were the collection volumes at times n and n − 1, respectively.

### 2.5. Cell Culture

Human gastric epithelial cells (GES-1 cells) were kindly donated by China Pharmaceutical University Cell Center (Nanjing, China). GES-1 cells were cultured in DMEM medium with 10% fetal bovine serum (FBS), 100 U/mL penicillin, and 100 μg/mL streptomycin at 37 °C in 5% CO_2_.

#### 2.5.1. Flow Cytometry

The apoptosis of GES-1 cells was detected to evaluate in vitro efficacy against oxidative stress, using flow cytometry. H_2_O_2_ was selected as a stimulant to construct a cellular oxidative stress model [25]. The apoptosis-inducing capability was assessed with the Annexin V-FITC/PI Apoptosis Detection Kit (Beyotime, Shanghai). Briefly, GES-1 cells were seeded in 12-well plates at the density of 1 × 10^6^ cell/well, and incubated for 24 h in a cell incubator. Then, GES-1 cells were incubated with different formulations (Bai, SA/PASP@CaCO_3_ blank hydrogel, and Bai/SA/PASP@CaCO_3_ hydrogels) at a Bai concentration of 100 μM for 2 h. Next, except for the control group, cells in all other groups were stimulated with H_2_O_2_ (400 μM) for 24 h. Next, the culture medium was discarded and GES-1 cells were washed three times with PBS. Afterwards, the cells were digested using trypsin, and washed three times. Finally, the cells were processed according to the procedure of the AnnexinV-FITC/PI assay kit, and the apoptosis of GES-1 cells was measured using flow cytometry (Beckman, Brea, CA, USA).

#### 2.5.2. Western Blot (WB) Assay

WB was used to detect the expressions of proteins related to the NRF2/HO-1 signaling pathway, in order to investigate the potential mechanisms. GES-1 cells were seeded in 6-well plates at a density of 1 × 10^7^ cells/well, and cultured in a cell culture incubator for 24 h. GES-1 cells were incubated with different formulations (Bai, SA/PASP@CaCO_3_ blank hydrogel, and Bai/SA/PASP@CaCO_3_ hydrogel) at a Bai concentration of 100 μM for 2 h. Subsequently, except for the control group, cells in all other groups were stimulated with H_2_O_2_ (400 μM) for 24 h. Then, GES-1 cells were digested using trypsin and lysed in RIPA lysis solution. The total protein concentration was determined using the BCA kit. Next, 30 μg of protein was separated using 10% SDS-PAGE and transferred to PVDF membranes. The membranes were blocked with 5% nonfat milk for 2 h, and treated with the following antibodies overnight at 4 °C: Nuclear factor erythroid 2 (NRF-2) (1:1000), Heme oxygenase-1 (HO-1) (1:1000), Jun N-terminal kinase (JNK) (1:1000), phospho-JNK (p-JNK) (1:1000), cleaved caspase-3 (1:1000), Bcl-2 associated X (BAX) (1:1000), and B cell lymphoma-2 (Bcl-2) (1:1000). After washing the membranes three times with Tris-buffered saline with Tween-20 (TBST), they were treated with horseradish peroxidase-conjugated secondary antibodies (1:5000) at room temperature for 2 h. Finally, the bands were detected using ECL luminous fluid, and observed with a gel imaging system.

#### 2.5.3. Measurement of SOD, CAT, MDA, and GSH Levels In Vitro

To examine the in vitro inhibition to oxidative stress of Bai, GES-1 cells were seeded in 6-well plates at a density of 1 × 10^7^ cells/well, and cultured in a cell culture incubator for 24 h. GES-1 cells were incubated with different formulations (Bai, SA/PASP@CaCO_3_ blank hydrogel, and Bai/SA/PASP@CaCO_3_ hydrogel) at a Bai concentration of 100 μM for 2 h. Afterwards, except for the control group, cells in all other groups were stimulated with H_2_O_2_ (400 μM) for 24 h. Then, cells were digested using trypsin, and GES-1 cells were lysed in RIPA lysis solution. The levels of SOD, CAT, MDA, and GSH in GES-1 cells were detected through assay kits, according to the manufacturer’s instructions (Nanjing Jiancheng Bioengineering Institute Co., Ltd., Nanjing, China).

### 2.6. Animal Experiments

Sprague Dawley rats weighing between 180~200 g were treated. The animal experiment was conducted under the guidelines for the care and use of the Experimental Animal Center of Nantong University, and the protocol was also approved by the Ethics Committee.

#### 2.6.1. Ethanol-Induced Acute Gastric Injury

The acute gastric injury model was induced by gavage of ethanol [26]. Briefly, rats were randomly divided into five groups (*n* = 9): control, model, Bai, PASP@CaCO_3_ blank hydrogel, and Bai/PASP@CaCO_3_ hydrogel groups. First, the groups of Bai and Bai/PASP@CaCO_3_ hydrogel were treated using gavage with the corresponding preparations containing 50 mg/kg Bai. The blank hydrogel group was given the same amount of hydrogel as the Bai/PASP@CaCO_3_ hydrogel group, while the control and model groups were given equal amounts of saline by gavage. The therapeutic agents in each group were given in advance, and anhydrous ethanol (1 mL/each) was given by gavage 1 h later. After another 1 h, the rats were anesthetized and sacrificed; gastric tissues were collected to examine the ulcerations, and further evaluated with H&E staining.

#### 2.6.2. Acetic Acid-Induced Chronic Gastric Ulcer

The chronic gastric ulcer animal model was induced by glacial acetic acid [27]. Rats were randomly divided into five groups (*n* = 9): control, model, Bai, PASP@CaCO_3_ blank hydrogel, and Bai/PASP@CaCO_3_ hydrogel groups. Rats were anesthetized with 4% chloral hydrate, and their abdomens were opened via a midline incision below the xiphoid, 15 min later. Next, the submucosal layer of the anterior or posterior wall of the glandular stomach was injected with 0.03 mL of acetic acid. After the peritoneal cavity was flushed with antibiotics, the abdomen was sutured, layer by layer. The rats of the control group underwent the surgical procedure with the application of saline instead of acetic acid. Treatment began 24 h after surgery. The control group and model group were given the same amount of saline, while the groups of Bai and Bai/PASP@CaCO_3_ hydrogel were given the corresponding preparations containing 50 mg/kg Bai via gavage. The frequency of dosing was once a day for three consecutive days, and the rats were sacrificed on postoperative day 4 when the ulcers were most severe. Then, the stomach was completely excised and opened along a greater curvature to examine the ulcer, and further stained with H&E for histopathological analysis.

Meanwhile, WB was adopted to detect the expressions of proteins related to the NRF2/HO-1 signaling pathway in gastric tissues. The gastric tissues that were removed above were homogenized and lysed in RIPA. The following operation procedures were the same as Section 2.5.2. In addition, the levels of SOD, CAT, MDA, and GSH in gastric tissues were detected using the same operation as described in Section 2.5.3.

### 2.7. Data Analysis

The experimental results were expressed as mean ± SD. All statistical analyses were conducted with Student’s *t*-test or one-way ANOVA, and a value of *p* < 0.05 was considered to be statistically significant.

## 3. Results and Discussion

### 3.1. Preparation and Characterization of PASP@CaCO_3_ NPs

**Figure 1:**
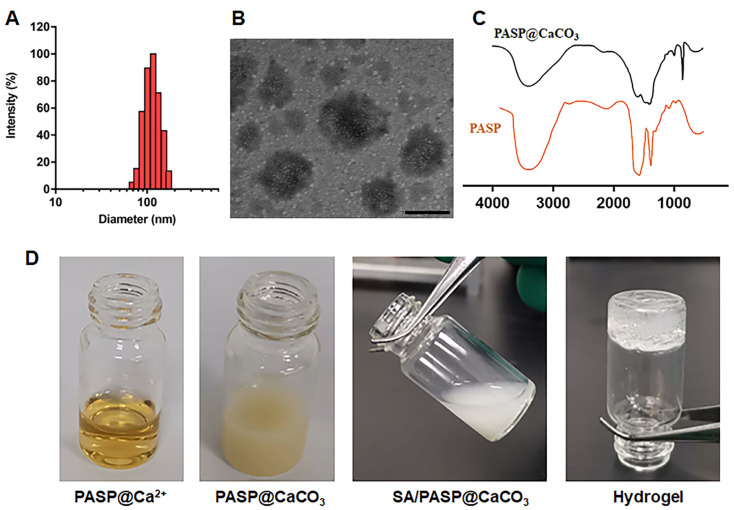
(**A**) DLS analysis of PASP@CaCO_3_ NPs. (**B**) TEM of PASP@CaCO_3_ NPs (scale bar: 100 nm). (**C**) FT-IR analysis of PASP and PASP@CaCO_3_ NPs. (**D**) The gelation process of SA/PASP@CaCO_3_ NPs.

PASP@CaCO_3_ nanoparticles were prepared using a one-pot strategy [24]. PASP, with its carboxyl long-chain structure as a green scale inhibitor, can bind to metal cations through strong electrostatic interactions. Meanwhile, the long chains surrounded by the exterior can stop the aggregation and growth of scale [28,29]. In this design, PASP was selected as a controller to prepare PASP@CaCO_3_ NPs with uniform particle size. As shown in Figure 1A, the hydrodynamic particle size of PASP@CaCO_3_ NPs was about 110.32 ± 3.21 nm, and the polydispersity index (PDI) was 0.234 ± 0.06, illustrating good dispersion capability. Meanwhile, the zeta potential of nanoparticles was approximately −25.56 ± 3.24 mV, showing lower cytotoxicity. The morphology of PASP@CaCO_3_ NPs was further detected with TEM. As exhibited in Figure 1B, it was clearly observed that PASP@CaCO_3_ NPs were homogeneous in size, spherical in shape, and showed good dispersion (Figure 1B). FT-IR spectroscopy was used to confirm the structure of PASP@CaCO_3_ and PASP. As depicted in Figure 1C, the observed absorbance band at 864 cm^−1^ corresponded to the out-of-plane bending of the amorphous structure for typical CaCO_3_. Additionally, the peaks of a doublet band at 1413 and 1459 cm^−1^ were also characteristic for the asymmetric stretching vibrations of carbonates. The stretching vibrations band at 3422 cm^−1^ was the typical peak of PASP, which also presented in PASP@CaCO_3_. Hence, the above results confirmed the successful preparation of PASP@CaCO_3_ NPs.

### 3.2. Preparation and Characterization of SA/PASP@CaCO_3_ In Situ Hydrogel System

#### 3.2.1. Gelation Study of SA/PASP@CaCO_3_ Hydrogel System

Next, we examined the gelation ability of the SA/PASP@CaCO_3_ delivery system. The preparation of SA/PASP@CaCO_3_ hydrogels was photographed and recorded, as shown in Figure 1D. It could be clearly seen that the prepared SA/PASP@CaCO_3_ pre-gel solution was in solution state, and when pH 1.2 HCl solution was added, the encapsulated CaCO_3_ dissolved in time to effectively release the cross-linker Ca^2+^, thus forming a uniform hydrogel. This demonstrated that the prepared SA/PASP@CaCO_3_ delivery system could form hydrogels in response to gastric acid pH.

**Figure 2:**
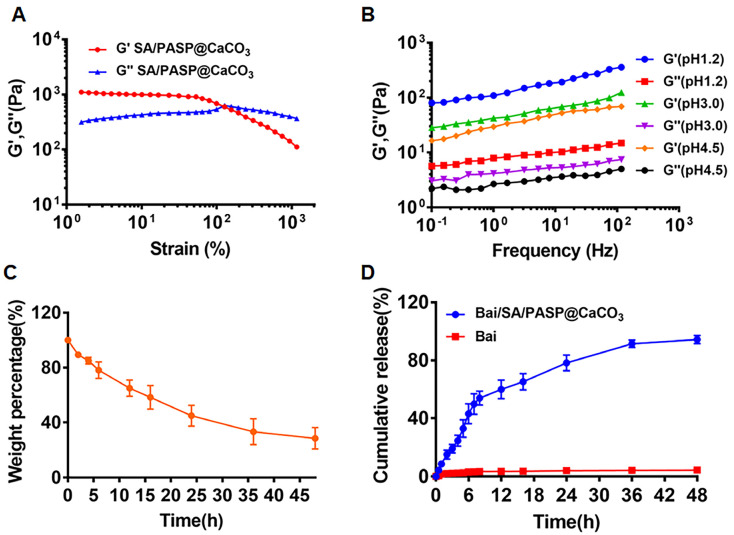
(**A**) The gelation comparison of the SA/PASP@CaCO_3_ hydrogels. (**B**) Images show the moduli of the SA/PASP@CaCO_3_ in different pH solutions. (**C**) The weight percentage variation of SA/PASP@CaCO_3_ in pH 1.2 HCl solution. (**D**) In vitro release profile. The data are expressed as mean ± SD (*n* = 3).

Viscoelasticity studies were performed to assess the mechanical properties of the SA/PASP@CaCO_3_ hydrogels. It is well known that the loss modulus (G″) of aqueous solutions is higher than the energy storage modulus (G′), while the energy storage modulus (G′) of hydrogels is greater than the loss modulus (G″) [30]. The good mechanical strength of the hydrogel allows it to remain intact in the gastric lining, hence reducing rapid leakage of the drug. As shown in Figure 2A, the strain amplitude sweep ranged from 1% to 1000%. The curves of G′ and G″ crossed at the strain of 127.6%, which was the critical strain value that destroyed the gel network and converted it into a solution state. Meanwhile, the modulus remained steady from 1% to 10%, and the G′ began to decrease when the strain was above 10%. Therefore, 2% of the oscillating strain was selected for the subsequent experiment.

#### 3.2.2. Effect of pH Variations on Gelation

Under normal conditions, the pH of gastric juice is generally between 0.9 and 1.8; meanwhile, due to feeding or other pathological reasons, gastric juice becomes diluted, and the pH rises to about 3.5. Therefore, pH values of 3.0 and 4.5 were selected to examine the modulus of the designed hydrogels, in order to evaluate the gelation ability of the SA/PASP@CaCO_3_ pre-gel solution when gastric juice was diluted. As illustrated in Figure 2B, the G′ of SA/PASP@CaCO_3_ hydrogels was larger than G″ under different pH conditions, presenting the classical hydrogel characteristics, indicating that the SA/PASP@CaCO_3_ hydrogel pre-gel solution could gel effectively under different intragastric pH conditions.

#### 3.2.3. Weight Loss Assay of SA/PASP@CaCO_3_ Hydrogel

The weight loss experiments were performed to examine in vitro erosion and degradation of SA/PASP@CaCO_3_ hydrogels. As mentioned above, the sustainable release of Ca^2+^ facilitates the maintenance of mechanical strength of the hydrogel to slow down the rate of hydrogel erosion and degradation [24]. As shown in Figure 2C, the weight of SA/PASP@CaCO_3_ maintained a slow decrease, and remained at 44.9% after 24 h; it was still 28.5%, even after 48 h. Overall, the above results indicated that SA/PASP@CaCO_3_ hydrogels could better maintain the network structure after the introduction of Ca^2+^, which also facilitated the sustained release of drugs.

#### 3.2.4. In Vitro Drug Release of Bai from Bai/SA/PASP@CaCO_3_ Hydrogels

First, drug loading and encapsulation efficiency of Bai in Bai/SA/PASP@CaCO_3_ hydrogels were detected using HPLC. The drug loading of Bai was 8.65 ± 0.98%, and the encapsulation efficiency of Bai was 92.45 ± 1.22%. Then, the drug release-time kinetics of the in situ hydrogel system was conducted in gastrointestinal simulation solution. As shown in Figure 2D, the cumulative release of Bai was less than 5% within 48 h. In contrast, Bai in Bai/SA/PASP@CaCO_3_ hydrogels released only 15% at 2 h and 49% at 7 h, exhibiting a significant sustained release characteristic. Many factors may have contributed to the sustained release behavior, including the continuous release of Ca^2+^, which promoted the cross-linking of the hydrogel network, and drugs being encapsulated in the porous hydrogel network. Accordingly, the designed Bai/SA/PASP@CaCO_3_ hydrogel system could release Bai continuously in the gastric, effectively avoiding the sudden release of Bai and increasing the absorption of Bai in the gastric mucosa.

### 3.3. Cell Experiments

#### 3.3.1. The Apoptosis Assay of GES-1 Cells

**Figure 3:**
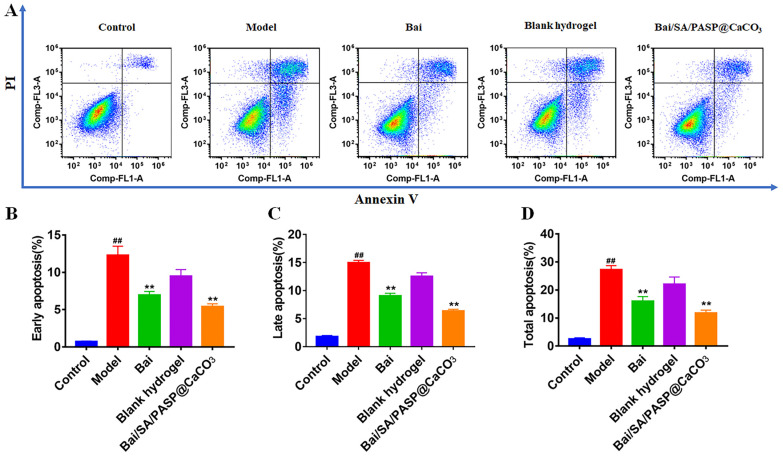
(**A**) Apoptosis rate of GES-1 cells was assessed with Annexin V-FITC/PI staining, and analyzed using flow cytometry. Quantitative analysis of early apoptosis (**B**), late apoptosis (**C**), and total apoptosis (**D**). Significant difference from the control group: ^##^ *p* < 0.01; Significant difference from the model group: ** *p* < 0.01.

Oxidative stress can lead to apoptosis of gastric epithelial cells [6]. To investigate the inhibitory effect of different preparations on oxidative stress, the apoptosis-inducing capability of different preparations was quantified via the Annexin V-FITC/PI method. Here, H_2_O_2_ was selected as a stimulant to construct a cellular oxidative stress model [25]. As shown in Figure 3, H_2_O_2_ effectively induced apoptosis of GES-1 cells and affected the distribution of cell scatter, indicating successful construction of the model. Meanwhile, the apoptotic rate of the control group was e negligible. In contrast, after treatment with Bai, cell apoptosis significantly decreased, and the percentages of early and late apoptotic cells of the Bai group were about 6.97% and 9.06%, respectively. Meanwhile, the apoptotic effect further decreased after treatment with Bai/SA/PASP@CaCO_3_ in situ hydrogels, and the proportions of early and late apoptotic cells were 5.42% and 6.35%, respectively. Accordingly, the above results all directly confirmed that Bai/SA/PASP@CaCO_3_ in situ hydrogel played an irreplaceable role in the protective effect on gastric mucosal epithelial cells.

#### 3.3.2. Western Blot (WB) Assay

**Figure 4:**
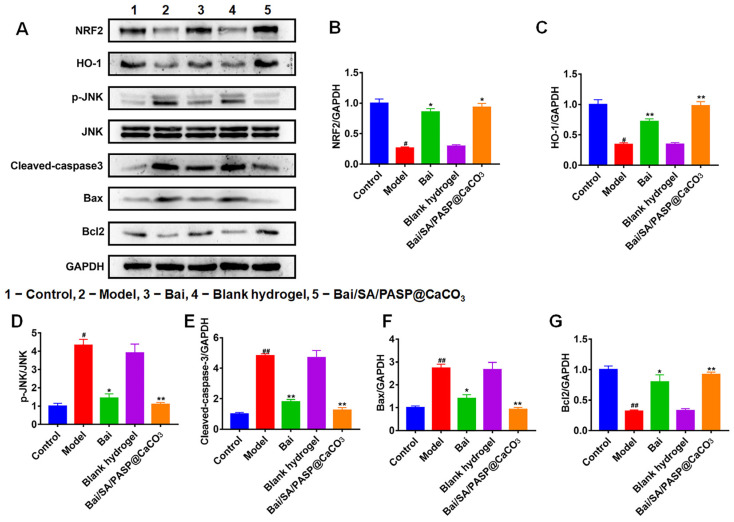
(**A**) The expressions of NRF2/HO-1 signaling related proteins on GES-1 cells after treated with Bai/SA/PASP@CaCO_3_ hydrogel. Quantitative analysis of NRF2 (**B**), HO-1 (**C**), p-JNK (**D**), Cleaved-caspase-3 (**E**), Bax (**F**), and Bcl2 (**G**) by Image J, according to Section A. Significant difference from the control group: ^#^ *p* < 0.05, ^##^ *p* < 0.01. Significant differences from the model group: * *p* < 0.05, ** *p* < 0.01.

NRF2/HO-1 signaling had a vital role in regulating apoptosis induced by oxidative stress [31]. JNK activation contributed to gastric mucosal epithelial cell apoptosis, and treatment with JNK inhibitor SP600125 effectively inhibited cellular apoptosis [32]. In this study, we detected the expression of NRF2/HO-1 signaling-related proteins, and also detected the level of apoptosis-related proteins p-JNK, cleaved-caspase3, Bax, and Bcl2. As shown in Figure 4, H_2_O_2_ stimulation significantly reduced the expression of NFR2, HO-1, and Bcl2, while increasing p-JNK, cleaved-caspase-3, and Bax levels in GES-1 cells. Meanwhile, both Bai and Bai/SA/PASP@CaCO_3_ significantly increased the levels of NFR2, HO-1, and Bcl2 proteins, reduced levels of p-JNK, cleaved-caspase-3, and Bax. In contrast, the expression levels of NFR2, HO-1, and Bcl2 proteins significantly increased from the treatment of Bai and Bai/SA/PASP@CaCO_3_, while the expression levels of p-JNK, cleaved caspase-3 and Bax decreased. These results were consistent with the above apoptotic results, indicating that Bai could inhibit H_2_O_2_-induced apoptosis of GES-1 through the NRF2/HO-1 signaling pathway.

#### 3.3.3. Measurement of SOD, CAT, MDA, and GSH Levels In Vitro

**Figure 5:**
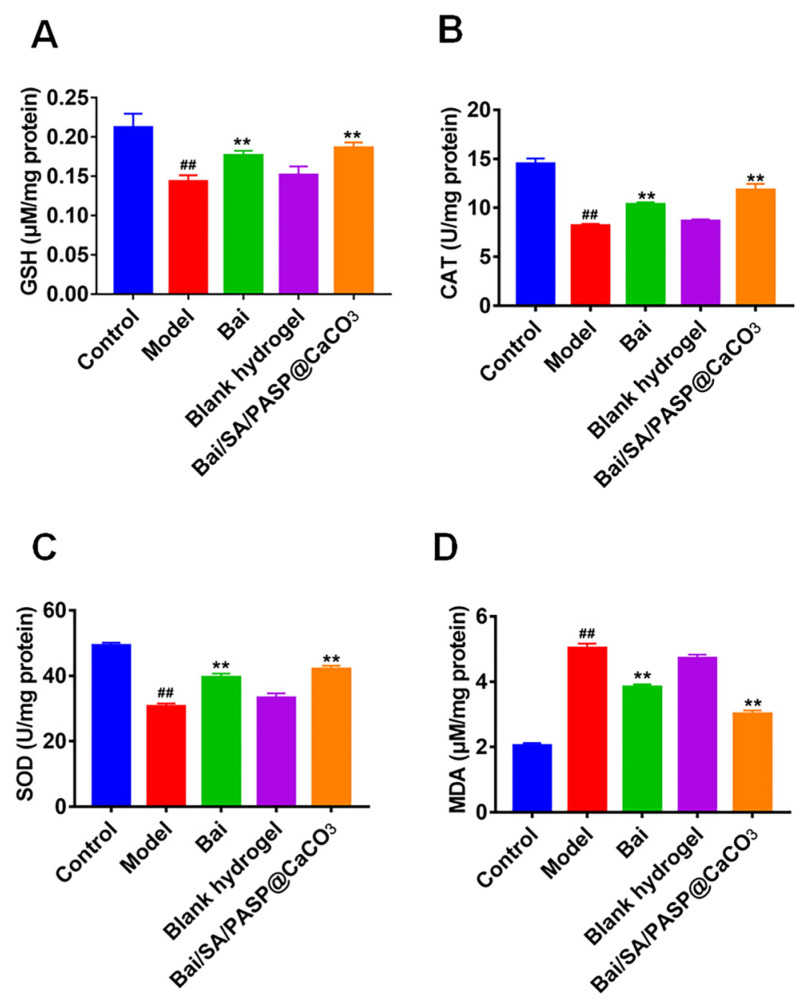
Effect of hydrogel on SOD (**A**), GSH (**B**), CAT (**C**), and MDA (**D**) activities in H_2_O_2_ -induced GES-1 cell injury in vitro. Data are presented as mean ± SD (*n* = 3). ^##^ *p* < 0.01 vs. the control group, and ** *p* < 0.01 vs. the model group.

Oxidative stress participated in the pathogenesis of GU, and the antioxidant drugs had a therapeutic effect on GU [33]. Previous studies have shown that antioxidant factors such as GSH, SOD, and CAT play an important role in regulating alcohol-induced gastric mucosal damage [30]. It is well known that H_2_O_2_ could induce oxidative stress injury in a variety of cells, such as GES-1 [25]. To investigate the anti-oxidative stress effects of Bai/SA/PASP@CaCO_3_ in vitro, we established an H_2_O_2_-induced GES-1 injury cell model. As shown in Figure 5, H_2_O_2_ stimulation significantly decreased the SOD and CAT activities and the GSH level; on the contrary, it increased the MDA level, while treatment with Bai and Bai/SA/PASP@CaCO_3_ markedly reversed these changes in oxidative stress-related markers. Importantly, the therapeutic effect of Bai/SA/PASP@CaCO_3_ was more obvious than that of Bai. Overall, the above results further proved that SA/PASP@CaCO_3_ could improve the inhibitory effect of Bai on oxidative stress.

### 3.4. Animal Experiments

#### 3.4.1. Effects of Bai/SA/PASP@CaCO_3_ Hydrogels on Gastric Mucosa Injury in Rats

**Figure 6:**
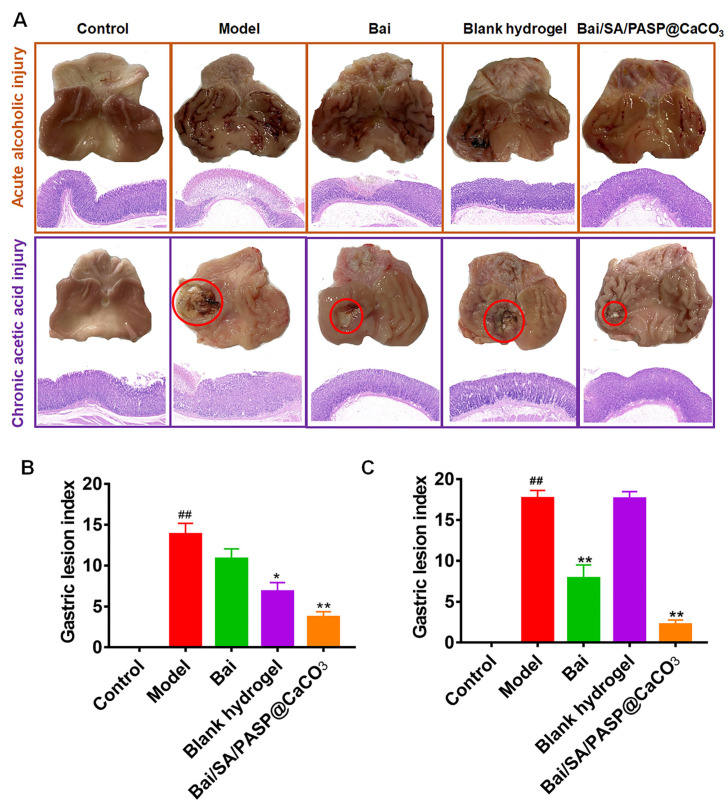
(**A**) Representative macroscopic images and histological assessment of the gastric tissue in ethanol-induced acute injury and acetic acid-induced chronic injury. Red circles represent the ulcers. Gastric lesion indexes of acute alcoholic injury (**B**) and chronic acetic acid injury (**C**). ^##^ *p* < 0.01 vs. the control group, and * *p* < 0.05 and ** *p* < 0.01 vs. the model group.

To evaluate the therapeutic effect of Bai/SA/PASP@CaCO_3_ on gastric ulcers, we established ethanol-induced acute and acetic acid-induced chronic gastric injury models. As presented in Figure 6, the surface of the gastric mucosa was smooth and intact, light pink in color, with no abnormal changes, such as congestion in the control group. Ethanol-induced hemorrhagic lesions in the acute GU model exhibited irregular dark red streaks of bleeding, diffuse gastric edema, mucosal erythema, and mucosal erosions, along with a high ulcer index (UI). The results of H&E staining uncovered that ethanol stimulation caused inflammatory infiltration, loss of parietal cells, marked edema and severe bleeding in the lower part of the gastric mucosa. In the acetic acid-induced chronic GU model, deep ulcer craters were observed in the gastric tissues. Evaluation of the histological sections showed that gastric tissue injury manifested as penetrating ulceration, accompanied by destruction of gastric mucosa and muscle tissue, infiltration of leukocytes, and formation of edema. Pretreatment with blank hydrogel and Bai/SA/PASP@CaCO_3_ significantly attenuated ethanol-induced mucosal damage and hemorrhagic lesions, which was accompanied by a decrease in infiltration of neutrophils into the surface epithelium. Bai had no obvious therapeutic effect on ethanol-induced GU. After 3 days of administration with Bai and Bai/SA/PASP@CaCO_3_ under the condition of acetic acid stimulation, the size and depth of GUs were significantly reduced, and submucosal edema was relieved. Moreover, the gastric tissue was re-epithelialized, and granulation tissue formed, suggesting the healing phase of the wound had initiated. It is worth noting that the therapeutic effect of Bai/SA/PASP@CaCO_3_ was better than that of Bai alone. These results indicated that PASP@CaCO_3_ facilitated the therapeutic effect of Bai for GU.

#### 3.4.2. Effects of Bai/SA/PASP@CaCO_3_ Hydrogels on Expressions of NRF2/HO-1 Signaling-Related Proteins

**Figure 7:**
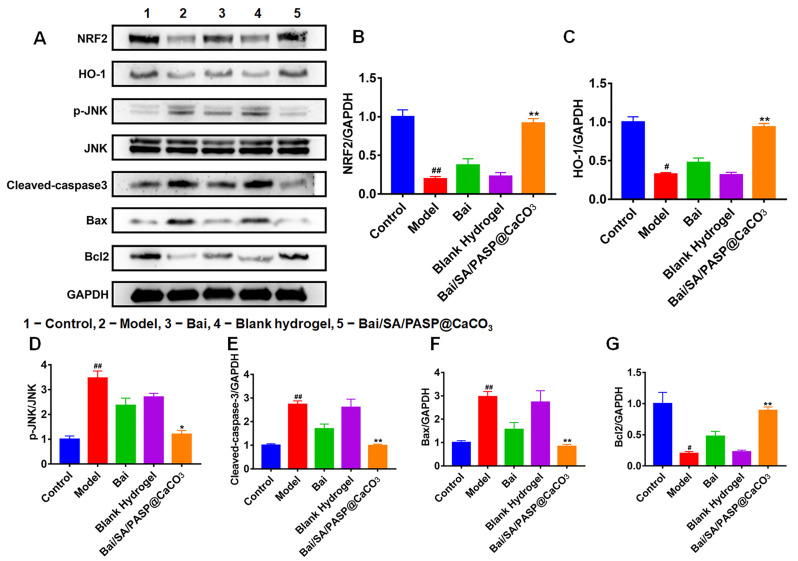
(**A**) Expressions of NRF2/HO-1 signaling-related proteins in rat stomach tissues after being treated with different therapeutic agents. Quantitative analysis of NRF2 (**B**), HO-1 (**C**), p-JNK (**D**), Cleaved-caspase-3 (**E**), Bax (**F**), and Bcl2 (**G**) by Image J, according to Section A. Significant difference from the control group: ^#^ *p*< 0.05, ^##^ *p* < 0.01. Significant differences from the model group: * *p* < 0.05, ** *p* < 0.01.

Meanwhile, we detected the expression levels of NRF2/HO-1 signaling-related proteins and apoptosis-related proteins p-JNK, cleaved-caspase3, Bax, and Bcl2 in gastric tissues. As shown in Figure 7, acetic acid stimulation significantly decreased the expressions of NFR2, HO-1, and Bcl2 in the gastric tissues of rats, while enhancing the expression levels of p-JNK, cleavage-caspase-3, and Bax. However, a significant reversal of the expressions of these proteins was observed after treatment with Bai/SA/PASP@CaCO_3_. Although the expression levels of these proteins were altered by Bai in gastric tissues, these changes were not significantly different compared to those in the model group. These results suggested that Bai-loaded SA/PASP@CaCO_3_ hydrogels effectively inhibited the oxidative stress in GU, and thus inhibited the apoptosis of gastric mucosal cells.

#### 3.4.3. Effects of Bai/SA/PASP@CaCO_3_ Hydrogels on Antioxidant Capacity

**Figure 8:**
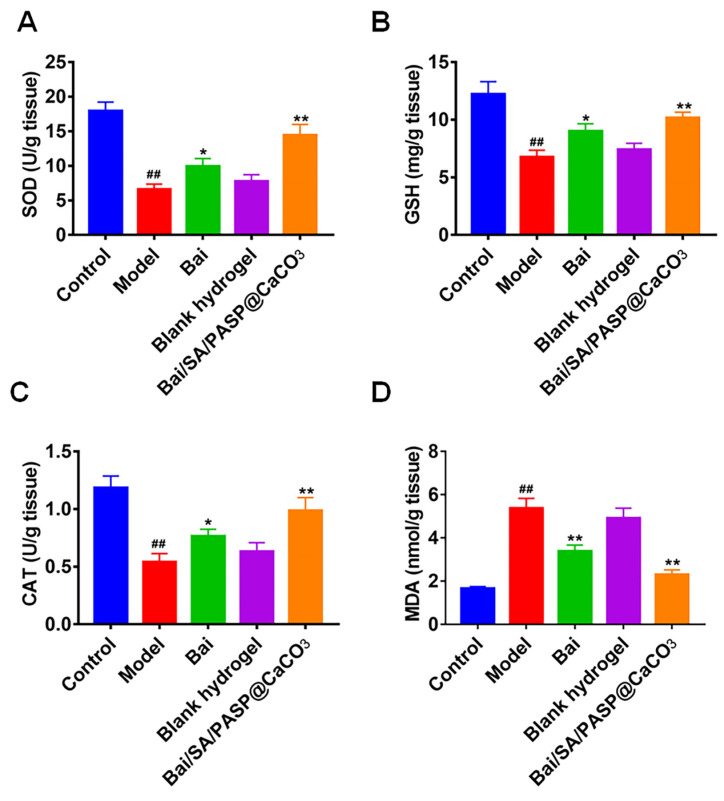
Effect of hydrogel on SOD (**A**), GSH (**B**), CAT (**C**), and MDA (**D**) activities in acetic acid-induced chronic gastric injury in vivo. Data are presented as mean ± SD (*n* = 3). ^##^ *p* < 0.01 vs. the control group, and * *p* < 0.05 and ** *p* < 0.01 vs. the model group.

We measured the expressions of CAT, SOD, GSH, and MDA, which were the most important indicators of oxidative stress in vivo. As shown in Figure 8, acetic acid stimulation triggered oxidative stress damage in gastric tissues by affecting the activities of SOD and CAT, as well as the levels of MDA and GSH. Acetic acid significantly decreased the activities of SOD and CAT as well as GSH levels in gastric tissues, and conversely increased the level of MDA. In contrast, treatment with Bai and Bai/SA/PASP@CaCO_3_ showed a significant reversal of these above changes. More importantly, the anti-oxidative stress effect of Bai/SA/PASP@CaCO_3_ was significantly different from that of Bai, indicating a superior anti-oxidative stress effect that was consistent with the experimental results in vitro.

## 4. Conclusions

In summary, we designed a gastric pH-responsive hydrogel (SA/PASP@CaCO_3_) for oral delivery of Bai for the treatment of GU. The drug-loaded in-suite hydrogel exhibited sustained release behavior and excellent biodegradability in vitro. Bai/SA/PASP@CaCO_3_ alleviated ethanol- and acetic acid-induced gastric ulcers. Moreover, Bai/SA/PASP@CaCO_3_ inhibited oxidative stress and apoptosis by regulating the NRF2/HO-1 signaling pathway, both in vivo and in vitro. Importantly, the anti-gastric ulcer effect of Bai/SA/PASP@CaCO_3_ hydrogel was better than that of Bai alone. Therefore, Bai/SA/PASP@CaCO_3_ hydrogel may serve as an oral drug delivery system, with potential applications for the clinical treatment of gastric ulcers.

## Data Availability

Not applicable.
